# Expert Evaluation of Strategies to Modernize Newborn Screening in the United States

**DOI:** 10.1001/jamanetworkopen.2021.40998

**Published:** 2021-12-29

**Authors:** Donald B. Bailey, Katherine Ackerman Porter, Sara M. Andrews, Melissa Raspa, Angela Y. Gwaltney, Holly L. Peay

**Affiliations:** 1 RTI International, Research Triangle Park, North Carolina

## Abstract

**Importance:**

Novel therapies, including cell and gene therapies, can radically improve outcomes among patients with rare disorders, especially if provided early. Newborn screening (NBS) could support early access to novel therapies, but the speed of new therapy development is a disruptive event for which the public health NBS system and state newborn screening programs are unprepared.

**Objective:**

To identify and evaluate possible solutions for modernizing NBS.

**Design, Setting, and Participants:**

In this survey study, NBS experts representing clinical research, federal or state advisory boards, patient advocacy groups, industry, or state laboratories completed an online survey in which they considered 20 potential solutions for modernizing NBS and rated each.

**Exposures:**

Participants considered 20 potential solutions in the 5 following domains: (1) timeliness of disorder review, (2) alternative mechanisms to offer screening for new disorders not currently part of NBS, (3) expanded data collection, (4) support for states, and (5) emerging methods of screening and their consequences.

**Main Outcomes and Measures:**

Mean ratings for each solution on efficacy, acceptability, feasibility, and sustainability.

**Results:**

The survey was completed by 40 NBS experts (median [range] age, 54 [37-73] years; 22 [55.0%] women). Participants acknowledged that substantial change is needed to prepare the NBS system for rapid expansion of novel therapies; on a scale of 0 (no change) to 10 (extensive change), the median (range) score was 8 (2-10), with 18 respondents (45.0%) believing that the NBS would need many new components or an entirely new system to accommodate the changes. All solutions for modernization were considered potentially efficacious by at least 23 respondents (57.5%). The 2 most strongly endorsed were to establish mechanisms for cross-state data coordination for provisional disorders (38 respondents [95.0%]) and create a network of regional screening laboratories (36 [90.0%]). These were closely followed by aligning programs across federal agencies (35 [87.5%]), expanding funding for research (34 [85.0%]), expanding funding to states (34 [85.0%]), building capacity to identify genetic variants and an associated clinical database (34 [85.0%]), and conducting surveillance to study long-term outcomes (34 [85.0%]).

**Conclusions and Relevance:**

In this study, there was consensus among experts that NBS needs to change if the system is to be prepared for a rapid increase in transformative therapies. To our knowledge, this is the first systematic inventory of potential solutions for modernizing NBS and expert perceptions of each. The findings suggest that the modernization of NBS will require the integration of highly rated solutions, strategic planning, and coordination among multiple stakeholders.

## Key Points

**Question:**

What solutions do subject matter experts recommend to prepare newborn screening for a rapid increase in the number of transformative therapies that must be provided early in life?

**Findings:**

In this survey study, 40 experts in newborn screening evaluated 20 potential solutions. The highest rated solutions addressed cross-state variability, national harmonization, data needed for clinical and policy decisions, and support to expedite state implementation.

**Meaning:**

These findings suggest that a coordinated national vision that incorporates effective solutions will be needed for newborn screening to accommodate an increasing number of transformative therapies.

## Teaser

This survey study identifies and evaluates expert recommendations for modernizing newborn screening in preparation for rapid increases in the number of novel therapies that must be provided early in life.

## Introduction

Newborn screening (NBS) is a highly successful public health program.^1^ All US states, with guidance from the US Department of Health and Human Services (DHHS), screen for disorders for which there are clear benefits from early detection and treatment. DHHS recommends 35 core disorders as part of the Recommended Uniform Screening Panel (RUSP), informed by an evidence review by the Advisory Committee on Heritable Disorders in Newborns and Children (ACHDNC).^2^ Because NBS programs operate under state auspice, each state decides whether to add an RUSP disorder to its screening panel. Most states screen for at least 30 recommended disorders and are gradually implementing the remainder.^3,4^

Unfortunately, NBS does not have the capacity to keep pace with a new generation of transformative therapies.^5,6^ The ACHDNC has only recommended 6 new disorders to be added to the RUSP since it was established in 2006. At best, it would take nearly 2 years for a condition to be nominated, fully reviewed, and recommended by the secretary. After that, state implementation varies widely, sometimes taking 3 to 5 years or longer. For example, spinal muscular atrophy was first nominated in 2008, but it was not until 2018, after the benefits of nusinersen had been demonstrated, that it was approved for the RUSP. The number of transformative treatments is growing dramatically, with projections of at least 60 cell and gene therapy approvals by 2030^7^ and hundreds more under investigation.^8^ Disorders previously lacking treatments could soon have interventions that are curative or significantly disease modifying. Their efficacy almost certainly will be maximized if provided early, underscoring the necessity of early screening and diagnosis.^9^

As new treatments are being developed, patient advocacy groups are becoming more active at both the state and national level, arguing that as soon as a treatment becomes available for a particular condition, it should be added to the RUSP. However, a critical feature of NBS policy is that an effective treatment is necessary but insufficient to justify screening for virtually any disorder. Information is needed about natural history, screening methods, follow-up protocols, and long-term outcomes. Although novel therapies could be transformative, their clinical utility will be complicated because of variable penetrance and phenotypic heterogeneity, the lack of predictive biomarkers, and uncertainties about long-term benefits and risks.

Even when a disorder is added to the RUSP, wide variability exists in state implementation, which can take years.^10^ Families, patient advocacy groups, and clinicians are frustrated by what they consider an unnecessarily long process of decision-making and implementation, resulting in preventable death, disability, or a lifetime of needed care. Policy makers, on the other hand, are concerned about adding new disorders without adequate evidence. Additionally, most state NBS laboratories cannot add new disorders without legislative approval, funding, and time to ensure adequate implementation.

Despite these challenges, transformative therapy development will continue, and pressure to add treatable disorders to NBS panels will be substantial. An onslaught of new and more effective therapies will constitute a disruptive event for which NBS is unprepared, and modernization of the entire NBS system seems unavoidable. But how should this modernization be accomplished? Anticipating this scenario, we enlisted expert stakeholders to identify solutions to challenges facing NBS and rate their efficacy, feasibility, acceptability, and sustainability.

## Methods

Research activities were framed around the following scenario: “It is 2030—ten years from today. Thirty or more new transformative gene or cell therapies have been approved by the FDA to treat monogenic, non-oncology rare disorders. Please consider the following assumptions. These assumptions may not reflect future reality but will be useful to frame our upcoming group discussion.

- Each treats a different genetic disorder.
- Each has a valid screening assay that is not prohibitively costly.
- The therapies are curative or significantly disease modifying if given early in life, but much less or not effective if given later.
- The longer-term risks and duration of efficacy are unknown.
- Assume that the cost of the therapies will be completely covered by payers (eg, insurance, Medicaid).”

### Participants

We recruited prominent individuals from 5 groups: (1) NBS researchers and clinicians, (2) state NBS program leaders, (3) patient advocacy organizations, (4) federal or state advisory committees, and (5) pharmaceutical or diagnostic companies. Participants were nominated by the research team, augmented by suggestions from project funders. A total of 55 invitations were sent, with the goal of at least 8 representatives from each group. Participants were offered a $100 gift card. The study was determined exempt by the RTI institutional review board. All participants provided written informed consent. This study followed American Association for Public Opinion Research (AAPOR) reporting guideline.

Overall, 42 experts participated in the first 2 phases of the study, and 40 completed all 3 phases. All but 1 participant were from the United States, and all had considerable NBS expertise. Although we selected by group—9 researchers/clinicians, 8 from state laboratories, 10 from patient advocacy groups, 8 from advisory committees, and 7 from industry—many represented multiple perspectives (eg, a clinician also on an advisory group). Participants were not asked to represent 1 group but to draw on the totality of their experiences and perspectives.

### Study Design

The study had 3 phases. First, there was a prepanel survey. After reviewing informed consent information and submitting an online acknowledgment of consent to participate in the study at large, 42 participants completed an online survey, rating their expertise in NBS and the likelihood of NBS expansion. On a scale from 0 (not at all) to 10 (extremely), participants rated knowledge of cell and gene therapies, NBS, and RUSP review. On a scale from 0 (not at all) to 10 (highly), participants rated the likelihood of (1) FDA approval of 30 therapies in 10 years; (2) the addition of those disorders to the RUSP by 2030; and (3) state implementation within 3 years of RUSP approval.

Second, we conducted 5 multistakeholder expert panels with 7 to 10 participants per panel (December 2020 to January 2021). Each was led by an author (H.L.P) experienced in moderation, with at least 1 representative from each group. A semi-structured guide was used to examine challenges facing NBS and explore solutions, their feasibility, and steps to implementation. We conducted a rapid qualitative analysis of discussions to identify and categorize solutions proposed by participants. A detailed qualitative analysis of data from the expert panels is forthcoming (S.M.A., unpublished paper, 2021).

Third, we created a postpanel survey. Approximately 1 month after the panels (February 2021), we sent a second online survey, completed by 40 panelists (95.0%). They rated the magnitude of change required to permit the timely inclusion of 30 new conditions in NBS, on a scale from 0 (no change) to 10 (extensive change throughout the NBS system). Respondents also chose a preferred approach from the 4 following options: (1) retain the current NBS system and make all changes within it; (2) retain most aspects of the current NBS system and make changes within it, while also developing a small number of new system components; (3) develop many new system components while retaining only a modest portion of the current NBS system; or (4) develop an entirely new system. We presented 20 potential solutions derived from the panel discussions. Solutions were organized into 5 domains, as follows: (1) revise and improve timeliness of RUSP review, (2) create mechanisms to offer screening for conditions in addition to those on the RUSP, (3) accelerate and expand data collection to inform policy decisions and implementation, (4) help states expedite comprehensive implementation of screening for new disorders, and (5) evaluate emerging methods of screening and their consequences. Using a 5-point scale from strongly disagree to strongly agree, respondents rated each solution on 4 dimensions: the extent to which each solution is efficacious, acceptable, feasible, and sustainable. Respondents could provide open-ended comments after each solution.

### Data Analysis 

In this article, we report descriptive analysis of the postpanel survey data and an overview of the open-ended comments associated with each solution domain. Full descriptions of solutions are displayed in Table 1. Survey responses were aggregated and summarized quantitatively (range and percentage of respondents). To simplify response summaries, we combined ratings of agree and strongly agree to create a single agreement score per solution. Given the sample size and the exploratory nature of this work, statistical tests were not used to compare solutions or model factors related to solutions.

**Table 1.** Solution Domains and Survey Items Associated With Each

Domain	Solutions
Domain 1: revise and improve timeliness of RUSP review	Solution 1.1: greatly expand funding for the ACHDNC’s work and the evidence review process to allow for simultaneous review of multiple disorders. Solution 1.2: build a more robust system to guide and support advocates in preparing a complete and compelling RUSP nomination package. Solution 1.3: move from a condition-by-condition review model to reviewing “bundles” of disorders with similar features. For example, a multiplex panel of related disorders or multiple conditions with an FDA-approved therapy could be reviewed at the same time. Solution 1.4: revise the amount and type of evidence required to support net benefit. Examples of expanded net benefit could include information about patient preferences, earlier referral to care, earlier access to an FDA-approved therapy, benefits to families, or access to early intervention programs.
Domain 2: create mechanisms to offer screening for conditions in addition to those on the RUSP	Solution 2.1: establish a provisional RUSP, allowing for unconsented screening of conditions for which there is a greater degree of uncertainty regarding the appropriateness or utility of screening due to insufficient data available at the time of FDA approval of a treatment. This would include a required review after several years to address specific research and implementation questions before permanent addition to the RUSP. Solution 2.2: establish a voluntary RUSP, an optional panel of disorders for which there is moderate certainty of net benefit, but major questions remain about diagnostic, treatment, or follow-up protocols (thus requiring consent). Offer the voluntary RUSP as part of a coordinated national program with a strong research component. Solution 2.3: create a model of public-private partnership with multistakeholder engagement to inform and support current and expanded NBS.
Domain 3: accelerate and expand data collection to inform policy decisions and implementation	Solution 3.1: align federal programs across agencies to maximize coordinated data collection and sharing to inform policy. Solution 3.2: greatly expand and expedite competitive funding for research and pilot studies, both investigator-initiated proposals and federally directed requests for proposals addressing cross-cutting issues. Solution 3.3: establish mechanisms for coordination of protocols and data integration for multiple states to rapidly implement and test feasibility of screening for provisional disorders. Solution 3.4: establish a coordinated network of research centers to anticipate important research questions or rapidly scale up to answer questions the ACHDNC or nominators believe to be essential prior to any recommendation. Solution 3.5: establish a national center and database to coordinate, collect, and analyze natural history data (outcomes) for children identified by NBS, assess family well-being and economic burden, and determine whether and how the long-term benefits of NBS are equitably experienced across families and states.
Domain 4: help states expedite comprehensive implementation of NBS for new disorders	Solution 4.1: expand national capacity for individualized technical assistance to states based on state needs and priorities. Solution 4.2: massively expand and sustain federal funding for state-based screening, but with stronger national authority and clear criteria that must be met to receive funding (eg, the state must adopt new disorders within a defined period of time). Solution 4.3: create and fund a network of regional screening laboratories (either commercial or academic), with the expertise and equipment to support the laboratory screening needs of individual states. Solution 4.4: create state or regional models for follow-up that provide equitable access to highly specialized treatments. Solution 4.5: raise public awareness of NBS by launching a national and sustained public information campaign about the combined public health impact of rare conditions, benefits of NBS, and the opportunity costs of not screening.
Domain 5: evaluate emerging methods of screening and their consequences	Solution 5.1: develop ongoing national capacity to assure that screening identifies children who require treatment and minimizes the identification of children who will never need treatment. Solution 5.2: launch a robust line of coordinated research designed to test the feasibility and benefits of genetic and genomic approaches to identify newly treatable disorders in NBS. Solution 5.3: conduct a series of studies on the costs, consequences for families and the health care system, and public perceptions of (1) false-positive screening outcomes and (2) the need for long-term surveillance to determine when and if transformative treatments should be provided to babies identified through screening.

Abbreviations: ACHDNC, Advisory Committee on Heritable Disorders in Newborns and Children; FDA, US Food and Drug Administration; NBS, newborn screening; RUSP, Recommended Uniform Screening Panel.

## Results

Experts representing the 5 key stakeholder groups (clinical researchers, state or federal advisory committees, patient advocacy organizations, state laboratories, and industry) completed presurvey ratings of their knowledge of NBS and reaction to the study scenario and a follow-up survey rating each of 20 potential solutions for modernizing NBS in anticipation of new transformative therapies. The 40 participants had a median (range) age of 54 (37-73) years, and 22 (55.0%) were women (Table 2).

**Table 2.** Participant Demographic Characteristics

Characteristic	Participants, No. (%) (N = 40)
Age	
Median (range), y	54 (37-73)
75 y or older	2 (5.0)
Prefer not to answer	3 (7.5)
Gender	
Woman	22 (55.0)
Man	16 (40.0)
Other	2 (5.0)
Education level	
Professional degree	29 (72.5)
Master’s degree	4 (10.0)
Bachelor’s degree	7 (17.5)
Race	
White	34 (85.0)
Asian	3 (7.5)
Other^a^	3 (7.5)
Ethnicity	
Hispanic	2 (5.0)
Not Hispanic	38 (95.0)

^a^ Respondents were given the option of responding other if none of the listed categories were appropriate.

### Self-rated Knowledge of NBS and Reaction to the Scenario

Participants were moderately knowledgeable about novel therapies (median [range] score, 7 [0-10]) and highly knowledgeable about NBS (median [range] score, 9 [5-10]) and RUSP review (median [range] score, 9 [5-10]). They were moderately optimistic about FDA approval of 30 transformative therapies within 10 years (median [range] score, 6 [0-10]). Fewer believed that all 30 could be added to the RUSP by 2030 (median [range] score, 3 [0-8]), and even fewer believed that states could add 30 within 3 years of RUSP approval (median [range] score, 2 [0-9]).

### Overall Perceptions of Magnitude and Focus of Change

Respondents to the postpanel survey agreed that substantial changes would be needed to add 30 disorders within a decade (median [range] score, 8 [2-10]), with nearly half (18 [45.0%]) responding with a 9 or 10. They were divided in their preferred approach for improving NBS. Slightly more than half (22 [55.0%]) preferred to retain the current system and make all changes within it, while developing a small number of new components. Others (18 [45.0%]) felt that more substantial changes were needed, developing many new components or an entirely new system.

### Overall Ratings of Solutions

Ratings for each solution are displayed in Table 3. Across all solutions, a mean of 78.0% of respondents agreed they were potentially efficacious, with a range from 57.5% to 95.0%. A mean of 74.0% (range, 35.0%-92.5%) agreed that the solutions would be acceptable. However, they were less optimistic about feasibility (median [range], 54.0% [42.5%-80.0%]) and sustainability (median [range], 47.6% [30.0%-62.5%]).

**Table 3.** Expert Ratings of Agreement With 4 Dimensions for Each of 20 Potential Solutions

Solution	Respondents, No. (%)				
	Strongly disagree	Disagree	Unsure	Agree	Strongly agree
Domain 1.0: Revise and improve timeliness of RUSP review					
1.1 Greatly expand funding for ACHDNC					
Efficacious	1 (2.5)	3 (7.5)	3 (7.5)	18 (45.0)	15 (37.5)
Acceptable	1 (2.5)	3 (7.5)	4 (10.0)	18 (45.0)	14 (35.0)
Feasible	2 (5.0)	3 (7.5)	18 (45.0)	8 (20.0)	9 (22.5)
Sustainable	3 (7.5)	7 (17.5)	14 (35.0)	10 (25.0)	6 (15.0)
1.2 Support advocates to prepare nominations					
Efficacious	1 (2.5)	6 (15.0)	8 (20.0)	17 (42.5)	8 (20.0)
Acceptable	1 (2.5)	3 (7.5)	4 (10.0)	23 (57.5)	9 (22.5)
Feasible	0	4 (10.0)	4 (10.0)	26 (65.0)	6 (15.0)
Sustainable	0	8 (27.5)	11 (27.5)	14 (35.0)	7 (17.5)
1.3 Review bundles of disorders					
Efficacious	2 (5.0)	5 (12.5)	3 (7.5)	17 (42.5)	13 (32.5)
Acceptable	3 (7.5)	7 (17.5)	7 (17.5)	16 (40.0)	7 (17.5)
Feasible	3 (7.5)	6 (15.0)	11 (27.5)	11 (27.5)	9 (22.5)
Sustainable	2 (5.0)	5 (12.5)	10 (25.0)	16 (40.0)	7 (17.5)
1.4 Expand net benefit considerations					
Efficacious	1 (2.5)	6 (15.0)	6 (15.0)	17 (42.5)	10 (25.0)
Acceptable	1 (2.5)	6 (15.0)	9 (22.5)	17 (42.5)	7 (17.5)
Feasible	1 (2.5)	5 (12.5)	15 (37.5)	13 (32.5)	6 (15.0)
Sustainable	1 (2.5)	5 (12.5)	17 (42.5)	13 (32.5)	4 (10.0)
Domain 2.0: create mechanisms to offer screening for conditions in addition to those on the RUSP					
2.1 Establish a provisional RUSP					
Efficacious	5 (12.5)	7 (17.5)	5 (12.5)	14 (35.0)	9 (22.5)
Acceptable	8 (20.0)	12 (30.0)	6 (15.0)	13 (32.5)	1 (2.5)
Feasible	5 (12.5)	6 (15.0)	10 (25.0)	16 (40.0)	3 (7.5)
Sustainable	5 (12.5)	8 (20.0)	14 (35.0)	10 (25.0)	3 (7.5)
2.2 Establish a voluntary RUSP					
Efficacious	2 (5.0)	6 (15.0)	6 (15.0)	17 (42.5)	9 (22.5)
Acceptable	2 (5.0)	8 (20.0)	6 (15.0)	17 (42.5)	7 (17.5)
Feasible	4 (10.0)	7 (17.5)	12 (30.0)	14 (35.0)	3 (7.5)
Sustainable	4 (10.0)	7 (17.5)	17 (42.5)	10 (25.0)	2 (5.0)
2.3 Create public-private partnerships					
Efficacious	0	0	8 (20.0)	16 (40.0)	16 (40.0)
Acceptable	1 (2.5)	0	9 (22.5)	13 (32.5)	17 (42.5)
Feasible	1 (2.5)	1 (2.5)	8 (20.0)	16 (40.0)	14 (35.0)
Sustainable	0	2 (5.0)	14 (35.0)	12 (30.0)	12 (30.0)
Domain 3.0: accelerate and expand data collection to inform policy decisions and implementation					
3.1 Align programs across federal agencies					
Efficacious	0	5 (12.5)	0 (0)	10 (25.0)	25 (62.5)
Acceptable	0	3 (7.5)	1 (2.5)	14 (35.0)	22 (55.0)
Feasible	1 (2.5)	4 (10.0)	14 (35.0)	13 (32.5)	8 (20.0)
Sustainable	0	3 (7.5)	12 (30.0)	13 (32.5)	12 (30.0)
3.2 Expand funding for research					
Efficacious	0	2 (5.0)	4 (10.0)	21 (52.5)	13 (32.5)
Acceptable	0	1 (2.5)	2 (5.0)	22 (55.0)	15 (37.5)
Feasible	0	4 (10.0)	9 (22.5)	19 (47.5)	8 (20.0)
Sustainable	1 (2.5)	6 (15.0)	16 (40.0)	12 (30.0)	5 (12.5)
3.3 Establish mechanisms for cross-state data coordination for provisional disorders					
Efficacious	1 (2.5)	0	1 (2.5)	18 (45.0)	20 (50.0)
Acceptable	1 (2.5)	3 (7.5)	4 (10.0)	17 (42.5)	15 (37.5)
Feasible	2 (5.0)	6 (15.0)	12 (30.0)	11 (27.5)	9 (22.5)
Sustainable	1 (2.5)	2 (5.0)	15 (37.5)	14 (35.0)	8 (20.0)
3.4 Establish a network of research centers					
Efficacious	0	3 (7.5)	6 (15.0)	15 (37.5)	16 (40.0)
Acceptable	0	4 (10.0)	5 (12.5)	18 (45.0)	13 (32.5)
Feasible	0	5 (12.5)	11 (27.5)	14 (35.0)	10 (25.0)
Sustainable	0	5 (12.5)	13 (32.5)	14 (35.0)	8 (20.0)
3.5 Establish a national center for longitudinal follow-up					
Efficacious	0	4 (10.0)	3 (7.5)	10 (25.0)	23 (57.5)
Acceptable	0	4 (10.0)	3 (7.5)	18 (45.0)	15 (37.5)
Feasible	0	5 (12.5)	14 (35.0)	12 (30.0)	9 (22.5)
Sustainable	0	6 (15.0)	14 (35.0)	13 (32.5)	7 (17.5)
Domain 4.0: help states explore comprehensive implementation of newborn screening for new disorders					
4.1 Expand state technical assistance					
Efficacious	1 (2.5)	2 (5.0)	5 (12.5)	14 (35.0)	18 (45.0)
Acceptable	1 (2.5)	1 (2.5)	6 (15.0)	16 (40.0)	16 (40.0)
Feasible	2 (5.0)	4 (10.0)	11 (27.5)	17 (42.5)	6 (15.0)
Sustainable	1 (2.5)	5 (12.5)	16 (40.0)	12 (30.0)	6 (15.0)
4.2 Expand federal funding to states					
Efficacious	1 (2.5)	2 (5.0)	3 (7.5)	18 (45.0)	16 (40.0)
Acceptable	3 (7.5)	4 (10.0)	10 (25.0)	10 (25.0)	13 (32.5)
Feasible	2 (5.0)	5 (12.5)	15 (37.5)	11 (27.5)	7 (17.5)
Sustainable	2 (5.0)	7 (17.5)	16 (40.0)	9 (22.5)	6 (15.0)
4.3 Create network of regional screening laboratories					
Efficacious	1 (2.5)	0 (0)	3 (7.5)	20 (50.0)	16 (40.0)
Acceptable	1 (2.5)	4 (10.0)	8 (20.0)	16 (40.0)	11 (27.5)
Feasible	1 (2.5)	3 (7.5)	11 (27.5)	15 (37.5)	10 (25.0)
Sustainable	1 (2.5)	4 (10.0)	12 (30.0)	15 (37.5)	8 (20.0)
4.4 Create state or regional models for specialized treatment access					
Efficacious	3 (7.5)	2 (5.0)	3 (7.5)	12 (30.0)	20 (50.0)
Acceptable	1 (2.5)	4 (10.0)	4 (10.0)	14 (35.0)	17 (42.5)
Feasible	2 (5.0)	7 (17.5)	10 (25.0)	12 (30.0)	9 (22.5)
Sustainable	1 (2.5)	7 (17.5)	10 (25.0)	12 (30.0)	10 (25.0)
4.5 Launch national public awareness campaign about benefits of NBS					
Efficacious	0	7 (17.5)	10 (25.0)	7 (17.5)	16 (40.0)
Acceptable	0	3 (7.5)	5 (12.5)	11 (27.5)	21 (52.5)
Feasible	0	4 (10.0)	8 (20.0)	14 (35.0)	14 (35.0)
Sustainable	0	10 (25.0)	14 (35.0)	6 (15.0)	10 (25.0)
Domain 5.0: evaluate emerging methods of screening and their consequences					
5.1 Build national capacity to identify genetic variants and associated clinical database					
Efficacious	1 (2.5)	1 (2.5)	4 (10.0)	17 (42.5)	17 (42.5)
Acceptable	1 (2.5)	1 (2.5)	1 (2.5)	18 (45.0)	19 (47.5)
Feasible	1 (2.5)	3 (7.5)	16 (40.0)	14 (35.0)	6 (15.0)
Sustainable	1 (2.5)	2 (5.0)	16 (40.0)	13 (32.5)	8 (20.0)
5.2 Launch coordinated research to evaluate genetic and genomic screening approaches					
Efficacious	1 (2.5)	1 (2.5)	5 (12.5)	19 (47.5)	14 (35.0)
Acceptable	0	2 (5.0)	6 (15.0)	21 (52.5)	11 (27.5)
Feasible	0	2 (5.0)	11 (27.5)	18 (45.0)	9 (22.5)
Sustainable	0	5 (12.5)	16 (40.0)	13 (32.5)	6 (15.0)
5.3 Conduct series of studies on long-term consequences of false positives and surveillance of screen-positive cases					
Efficacious	2 (5.0)	3 (7.5)	1 (2.5)	16 (40.0)	18 (45.0)
Acceptable	2 (5.0)	1 (2.5)	2 (5.0)	21 (52.5)	14 (35.0)
Feasible	2 (5.0)	5 (12.5)	9 (22.5)	13 (32.5)	11 (27.5)
Sustainable	2 (5.0)	6 (15.0)	17 (42.5)	7 (17.5)	8 (20.0)

Abbreviations: ACHDNC, Advisory Committee on Heritable Disorders in Newborns and Children; NBS, newborn screening; RUSP, Recommended Uniform Screening Panel.

### Domain 1: Revise and Improve Timeliness of RUSP Review

Four solutions addressed the slow pace of ACHDNC review: (1.1) greatly expand funding for the ACHDNC, (1.2) support advocates to prepare nominations, (1.3) review bundles of disorders, and (1.4) expand criteria for what constitutes net benefit. The solution considered most efficacious was to expand funding to allow simultaneous review of multiple disorders (33 participants [82.5%]), followed by a related solution—review bundles of disorders at the same time (30 [75.0%]). Expanded funding was rated more acceptable (32 [80.0%]) than reviewing bundles (23 [57.5%]), but fewer than half rated either as feasible or sustainable.

Open-ended comments generally reflected the opinion that something must be done to expedite condition reviews, but there was less agreement on how that might be accomplished. Reviewing bundles of disorders elicited the most open-ended comments, generally reflecting concern that each disorder would have unique features that must be considered.

Participants moderately agreed that supporting advocates to prepare nominations would be efficacious (25 [62.5%]) and considered this solution acceptable (32 [80.0%]) and feasible (32 [80.0%]). Expanding criteria for net benefit also received moderate endorsement for efficacious (37 [67.5%]) and acceptable (24 [60.0%]), but less agreement that it would be feasible (19 [47.5%]) or sustainable (17 [42.5%]). One said, “Solution 1.4 is what we should strive for—that’s what would help the patients the most,” yet another said, “I do not think that expanding the definition of net benefit will speed up… the process.… [R]ather it would likely have just the opposite effect.” A third participant was concerned that expanding the definition of benefit might “risk states no longer regarding the RUSP so highly.”

### Domain 2: Create Mechanisms to Offer Screening for Conditions in Addition to the RUSP

Three solutions suggested alternative screening options prior to RUSP approval: (2.1) establish a provisional RUSP, (2.2) establish a voluntary RUSP, and (2.3) create public-private partnerships. The solution considered most efficacious was public-private partnerships to support expanded NBS (32 [80.0%]), which was also generally considered acceptable (30 [75.0%]) and feasible (30 [75.0%]), although with less certainty about sustainability (24 [60.0%]). Some were concerned about potential conflicts of interest if partners included diagnostic or pharmaceutical companies. Establishing a provisional RUSP (23 [57.5%] ) or a voluntary RUSP (26 [65.0%]) were considered less efficacious, with considerable uncertainty about feasibility and sustainability. Transformative therapies themselves are not likely to evoke radically different ethical issues or push NBS to change to a consented model, but a voluntary, 2-tier system would. Open-ended comments reflected concerns about how expensive and complicated it would be to set up these systems and the risks imposed to current NBS if research and consent processes were introduced.

### Domain 3: Accelerate and Expand Data Collection to Inform Policy and Implementation

Five solutions addressed the fact that even with an approved therapy, absence of critical data will impede rapid expansion: (3.1) align programs across federal agencies, (3.2) expand funding for research, (3.3) establish mechanisms for cross-state coordination for provisional disorders, (3.4) establish a network of research centers, and (3.5) establish a national center for longitudinal follow-up. All were considered potentially efficacious by most respondents. Solution 3.3 (cross-state coordination) was the highest rated in any domain, endorsed by 38 respondents (95.0%). All were generally considered acceptable, with 35 respondents (87.5%) supporting aligning programs across federal agencies and 34 [85.0%]) supporting expanding funding for research, but feasibility and sustainability reflected less certainty.

Open-ended comments generally reflected support for expanded data collection. As 1 participant said, “A coordinated national effort to harmonize data collection and facilitate pilot studies would be the single most effective way to enhance NBS in the future.” Several comments emphasized the need for a stronger federal role, but competing agency priorities was a barrier, as were states’ willingness or ability to collect and share data.

### Domain 4: Help States Expedite Comprehensive Implementation of Screening for New Disorders

Five solutions addressed state implementation: (4.1) expand state technical assistance, (4.2) expand federal funding to states, (4.3) create a network of regional screening laboratories, (4.4) create state or regional models for specialized treatment access, and (4.5) launch a national public awareness campaign about the benefits of NBS. Four were endorsed as likely efficacious by 80% or more respondents: expand state technical assistance (32 [80.0%]), expand federal funding to states (34 [85.0%]), create a network of regional screening laboratories (36 [90.0%]), and build models for specialized treatment access (32 [80.0%]). Although 85.0% supported expanding federal funding to states, only 15 (37.5%) believed such a program would be sustainable. A public awareness campaign was the lowest rated for efficacious across all domains but still was endorsed by 23 respondents (57.5%).

Some open-ended comments reflected concerns about state vs federal authority. One respondent said, “More mandates from the feds, even if funded, are not going to be swallowed very easily by many states”; in contrast another said, “In our current climate, a federal mandate to screen would be well-accepted by many states.” One participant suggested that commercializing NBS might be the answer, whereas another said, “It really seems that a regionalization approach is the best and most innovative solution.”

### Domain 5: Evaluate Emerging Methods of Screening and Their Consequences

Many conditions that could be treated by transformative therapies will need to be identified through genetic testing, and ultimately, next-generation sequencing could be used to multiplex many conditions at the same time. But sequencing can evoke a wide range of concerns, from the technical to the clinical, and most states have limited capacity for multiplexed genetic screening. Three solutions addressed concerns about disorder heterogeneity, uncertainty about who would need treatment, the need for long-term surveillance, and genomic screening: (5.1) build national capacity to identify genetic variants and an associated clinical database, (5.2) launch coordinated research to evaluate genetic and genomic screening approaches, and (5.3) conduct surveillance to study long-term outcomes for children and families. All 3 solutions were endorsed as efficacious by more than 80% of respondents, and all were similarly considered acceptable. Both building national capacity to identify genetic variants and an associated clinical database and conducting a series of studies on the long-term consequences of false-positive results and surveillance of screen-positive cases were supported by 34 respondents (85.0%), and only a few respondents expressed strong disagreement with these solutions.

## Discussion

Transformative therapies threaten to overwhelm state and national NBS capacity. Although the problems are well known,^11,12^ the solutions are less obvious. To address this limitation, we engaged expert NBS stakeholders to suggest and rate solutions. The findings provide policy-relevant information about the viability of solutions needed to prepare NBS for new therapies. Several observations are noteworthy.

First, substantial change is needed to prepare NBS for treatment advances. There was little optimism among participants that 30 new disorders could be added to the RUSP by 2030 or that states could add 30 within 3 years of RUSP approval. There was strong agreement that some form of modernization is essential. Participants varied in the extent to which they thought changes should be made within the existing NBS system or whether many new components are needed.

Second, all solutions for modernization were considered potentially efficacious by most respondents (>57.0%). The 2 most strongly endorsed were to establish mechanisms for cross-state data coordination for provisional disorders (95%) and create a network of regional screening labs (90.0%). These were closely followed by aligning programs across federal agencies (87.5%), expanding funding for research (85.0%), expanding funding to states (85.0%), building capacity to identify genetic variants and an associated clinical database (85.0%), and conducting surveillance to study long-term outcomes (85.0%).

Third, variability in perceived efficacy was evident across solutions and participants. This could be partly attributed to the fact that many solutions had multiple components. We did not compare variability across stakeholder groups because many participants had multiple affiliations. Variability in stakeholder perspectives will need to be addressed if any modernization initiative is to be successful.

NBS as we know it needs substantial reform. Ideally, this would lead to a well-coordinated and nimble national system. In reality, NBS is a complex and loosely interconnected set of independent state programs with many components, under the auspices of state health departments, guided by national recommendations, slowly evolving in response to forces outside of its control. The inevitability of new and more effective therapies is one such force for which the current system is unprepared, creating an urgency to quickly identify and implement workable solutions.

But modernizing NBS will require a clearly articulated strategy. Changing a well-established system after 60 years will not be easy, especially given the varying perspectives of state and federal governments, researchers, clinicians, industry representatives, and patient advocates. These stakeholders share the same goal: identify infants with serious health conditions and provide treatments as early as possible. But they vary considerably in auspice, history, resources, priorities, and responsibilities, and so it is not surprising that they do not speak with a common voice.

The highest rated solutions suggest critical steps. First, there is an urgent need for better data. A near-term goal should be coordinated efforts to integrate disparate data systems and conduct analyses to answer critical questions required for decision-making. In the meantime, work should begin to establish a more formal national data system driven by key overarching questions and nimble enough to respond quickly to needs for data. Second, there is an urgent need to help states expedite implementation. This will likely require a combination of national leadership, an infusion of federal funds, and perhaps regionalization of laboratory services.

Increased funding will be essential to enable critical decision-making, conduct evidence reviews, support pilot studies and research, harmonize and integrate data, support state implementation, and build regional screening labs. But a massive increase in funds without a coordinated strategic plan will be ineffective and potentially counterproductive. The vision for such a plan must be to bring the benefits of safe and effective treatments to infants more quickly. Building a next-generation NBS system to accomplish this vision will require extensive stakeholder engagement to create community consensus, a willingness of federal agencies to cooperate in implementing solutions, an intensive effort to harmonize state policies and resources, and ultimately national legislation.

### Limitations

Several limitations should be noted. We invited a nationally prominent group of participants, but the sample was nominated based on expertise and not selected to be representative. Although other suggestions came up during the panel, we focused on cross-cutting solutions, most of which included multiple components, to create a manageable survey. Third, although participants rated discrete solutions, we did not ask them to propose a combined package. Clearly, efficacy would be enhanced if multiple solutions were implemented in a complementary fashion.

## Conclusions

This survey study found that NBS experts recommended substantial changes to the current system to prepare for rapid increases in transformative therapies. These solutions included establishing mechanisms for cross-state data coordination for provisional disorders and creating a network of regional screening laboratories. The recommended changes will require national leadership and funding as well as further study of optimal ways to harmonize state policies and resources.
